# *Ambrosia* (ragweed) pollen — A growing aeroallergen of concern in South Africa

**DOI:** 10.1016/j.waojou.2024.101011

**Published:** 2024-12-02

**Authors:** Dorra Gharbi, Dilys Berman, Frank H. Neumann, Trevor Hill, Siyavuya Sidla, Sarel S. Cillers, Jurgens Staats, Nanike Esterhuizen, Linus Ajikah, Moteng E. Moseri, Lynne J. Quick, Erin Hilmer, Andri Van Aardt, Juanette John, Rebecca Garland, Jemma Finch, Werner Hoek, Marion Bamford, Riaz Y. Seedat, Ahmed I. Manjra, Jonny Peter

**Affiliations:** a Division of Allergology and Clinical Immunology, Department of Medicine, University of Cape Town, Cape Town, South Africa; b Allergy and Immunology Unit, Lung Institute, University of Cape Town, South Africa; c Unit for Environmental Sciences and Management, Faculty of Natural and Agricultural Science, North-West University, Potchefstroom, South Africa; d Discipline of Geography, University of KwaZulu-Natal, Pietermaritzburg, South Africa; e Department of Family Medicine, Faculty of Health Sciences, University of the Witwatersrand, Johannesburg, South Africa; f Department of Conservation Ecology and Entomology, University of Stellenbosch, Stellenbosch, South Africa; g Evolutionary Studies Institute and School of Geosciences, University of the Witwatersrand, Johannesburg, South Africa; h African Centre for Coastal Paleoscience, Nelson Mandela University, Gqeberha, South Africa; i Department of Plant Sciences, Faculty of Natural and Agricultural Sciences, University of the Free State, Bloemfontein, South Africa; j Smart Place, CSIR, Pretoria, South Africa; k Department of Geography, Geoinformatics and Meteorology, University of Pretoria, Pretoria, South Africa; l Department of Otorhinolaryngology, Gariep Mediclinic, Kimberley, South Africa; m Department of Otorhinolaryngology, Faculty of Health Sciences, University of the Free State, Bloemfontein, South Africa; n Hiway Medical Centre, Westville Hospital, Durban, South Africa

**Keywords:** *Ambrosia*, Bioaerosol monitoring, eDNA metabarcoding, Allergenicity, Southern Africa

## Abstract

**Background:**

Ragweed is an invasive, highly allergenic weed predicted to expand its habitat with warming global temperatures. Several *Ambrosia* species have been identified in South Africa for well over a century; however, its presence remained undetected by allergists and aerobiologists until the development of an extensive aerospora monitoring system across South African urban areas since 2019. This paper presents the inventory of preliminary investigation of the *Ambrosia* airborne pollen and the taxonomic identification of ragweed species.

**Methods:**

Burkard volumetric spore traps for collecting pollen samples are set up in 9 South African cities (Johannesburg, Cape Town, Pretoria, Kimberley, Durban, Potchefstroom, Ermelo, Bloemfontein, and Gqeberha). Light microscopic identification was combined with environmental DNA metabarcoding analysis to confirm the species level of airborne *Ambrosia* at selected monitoring stations. Ragweed sensitisation was examined in Cape Town between February 2019 and February 2024, using Allergy Xplorer (ALEX^2^) multicomponent allergen array.

**Results:**

*Ambrosia* pollen was detected in 5 aerobiological monitoring stations over the sampling period (Durban, Kimberley, Pretoria, Potchefstroom, Johannesburg). Periods of 4 consistent pollination years were observed in Kimberley (min: 1; max: 16 p.g/m^3^) and Durban (min: 26; max: 66 p.g/m^3^). In Pretoria, ragweed pollen was detected for 2 years (2020–2021; 2022–2023) with average total annuals (5-17 p.g/m^3^). A peak flowering period between March and April was observed in Potchefstroom, and several ragweed pollen peaks were present between the end of December and the beginning of May in Durban. The highest number of *Ambrosia* pollen grains was recorded in Potchefstroom, with 308 grains, and a maximum peak of 47 p.g/m^3^. eDNA metabarcoding confirmed the presence of *Ambrosia artemisiifolia* and *A.trifida* species. The overall prevalence of *Ambrosia*-sensitisation amongst 673 tests (age range 7–72 years) was 8.2% (55/673), with no significant difference in sensitisation patterns between age groups.

**Conclusion:**

Our study confirms the need to monitor the spread of ragweed, and an increasing awareness of Ambrosia as an allergen of concern in Southern Africa. Extension of aerobiological networks and testing for *Ambrosia* sensitisation across urban and rural sites will be required.

## Introduction

Ragweed (*Ambrosia* spp.) is an annual herbaceous weed belonging to the Asteraceae family, which has become a global spreading neophyte described in approximately 80 countries.^1^ In the Southern Hemisphere, *Ambrosia* spp. are represented by naturalized alien species in Southern Africa, alien invasive in Australia, and a few native species in Latin America.^1^

Approximately, 40 species are known, of which *Ambrosia trifida* L. (giant ragweed), *Ambrosia tenuifolia* Spreng. (slender or slim-leaf burr ragweed)*, Ambrosia psilostachya* DC. (Western or perennial ragweed) and *Ambrosia artemisiifolia* L. (common or short ragweed) are the most abundant worldwide.^2^ *Ambrosia* contains numerous pioneer species that inhabit a wide range of soils, habitat types, and climates^3^ and are distributed in ruderal fields, along roadsides, and in disturbed sites. The species are prolific seed producers and wind-pollinated and can produce high numbers of pollen grains per plant during the summer season.^4^ The grains are small (18–23 μm)^5^ and prone to long-distance transport.^6^

Ragweed is considered a species of concern in urban areas linked to traits of ecosystem disservices, specifically health issues in terms of allergenic pollen.^1^ *Ambrosia* pollen is detected in spores traps across many European countries (eg, France, Germany, Italy, Spain, Bulgaria, Hungary, Austria, Switzerland, Poland, Croatia, Czech Republic, Slovak Republic, Turkey, Sweden, and Romania).^7^ Common ragweed, native to North America, can also be found on other continents including Asia, eg, Seoul, Australia, South America, and Canada.^5^ In Africa, despite the presence of pollen monitoring networks in Morocco, Egypt, Tunisia, Nigeria, and Benin,^8^ no reports of *Ambrosia* pollen exist.

*Ambrosia* is becoming a weed of concern, reducing agriculture yields and an outdoor aeroallergen negatively impacting upon public health due to its highly allergenic pollen^7^ causing type I allergic reactions in late summer and early autumn.^9^ Ragweed pollen grains can induce different types of symptoms in sensitive people such as sneezing, itching, runny nose, and itchy eyes, and some individuals may develop asthma.^10^ The pollen allergens of *A. artemisiifolia*, *A. trifida*, and *A. psilostachya* are considered cross-reactive for skin testing and immunotherapy, it is generally believed that 1 species is sufficient.^11^ However, Asero et al^12^ in the northern area of Milan (widely invaded by *A. artemisiifolia*), revealed that approximately 50% of patients submitted to injection of a specific immunotherapy with *A. trifida* showed little or no clinical response. An excellent outcome was obtained only with *A. artemisiifolia-*specific immunotherapy.

### Ambrosia species in Africa

Ragweed species have become a global invasive since the 19th century.^5^ To date, *Ambrosia artemisiifolia* is the most common global *Ambrosia* species, with predominance in Europe, Asia, and Australia.^1^^,^^2^ In Africa, ragweed species were documented in studies linking Mediterranean and Sub-Saharan African flora analysis. Quezel and Santa^13^ reported the presence of alien naturalized species of *Ambrosia artemisiifolia* and *Ambrosia psilostachya* in Algeria. *Ambrosia* *artemisiifolia* was recorded in Egypt in 2002,^14^ and Libya,^15^ and *Ambrosia psilostachya* was recorded for the first time in Morocco in 1994.^16^ *Ambrosia artemisiifolia* is recorded from southern Africa (Botswana and Swaziland)^17^^,^^18^ and Madagascar.^19^ Kull et al^19^ mention that *Ambrosia maritima* was recorded as an introduced and naturalized taxon in Madagascar, and Macdonald et al^20^ reported the presence of *Ambrosia psilostachya* in Mauritius.

### Ambrosia DNA metabarcoding sequencing

Analysis of pollen grains by light microscopy is often challenging beyond the genus level due to common morphological features shared within Genera, Families, and even Orders; only pollen taxa are identifiable to the species level.^21^ Electron microscopy (SEM) can be applied to differentiate pollen to species level,^22^ and species identification in aerobiological studies can be solved using emerging molecular diagnostic methods. In the past few years, the application of DNA molecular research (called eDNA barcoding or metabarcoding) is becoming increasingly important as it provides information about plant traces and the biotic composition of entire ecosystems.^23^ Today, eDNA metabarcoding is applied to determine the taxonomic composition of airborne pollen exposure and provide information on the ecology of the atmospheric samples.^24^^,^^25^

Through DNA metabarcoding studies, one can demonstrate that the relative abundance of metabarcoding read counts correlates well with pollen concentrations identified under a light microscope.^26^ However, this correlation can depend on the species studied and the other species present in the mixture.^24^ A limited number of studies have investigated identifying and quantifying species composition using DNA metabarcoding of *Ambrosia* pollen collected with a 7-day volumetric trap.^24^ To date, no previous studies have identified specific taxa from an aerobiome of environmental air samples in Africa which makes our research novel.

### Ambrosia pollen monitoring in South Africa

Although many *Ambrosia* populations appear to have expanded in South African cities, the distribution of *Ambrosia* species has not been recorded in South Africa. Due to the need to understand the presence, distribution, and flowering time of endemic, exotic and invasive plants of South Africa (eg, *Ambrosia*), the South African Pollen Network (SAPNET; www.pollencount.co.za) was developed. SAPNET has been monitoring since August 2019 using a total of 7 standard pollen traps in 7 cities: Cape Town, Pretoria, Johannesburg, Kimberley, Durban, Bloemfontein, and Gqeberha.^27^ The network measurement was extended to Ermelo (August 2021) and Potchefstroom (December 2022) to collect aerospora and to develop monitoring and forecasting capacity for the risk of respiratory allergies.

In this study, based on 4 years of data, we applied the standard volumetric pollen sampling to evaluate the current spatiotemporal dynamics and diversity of *Ambrosia* spp. in South Africa. Secondly, we combined our first *Ambrosia* aerobiological comprehensive study with an eDNA metabarcoding pilot study to confirm *Ambrosia* spp present in our aerobiological samples.

## Material and methods

### Monitoring and analysis of pollen data

**Fig. 1:**
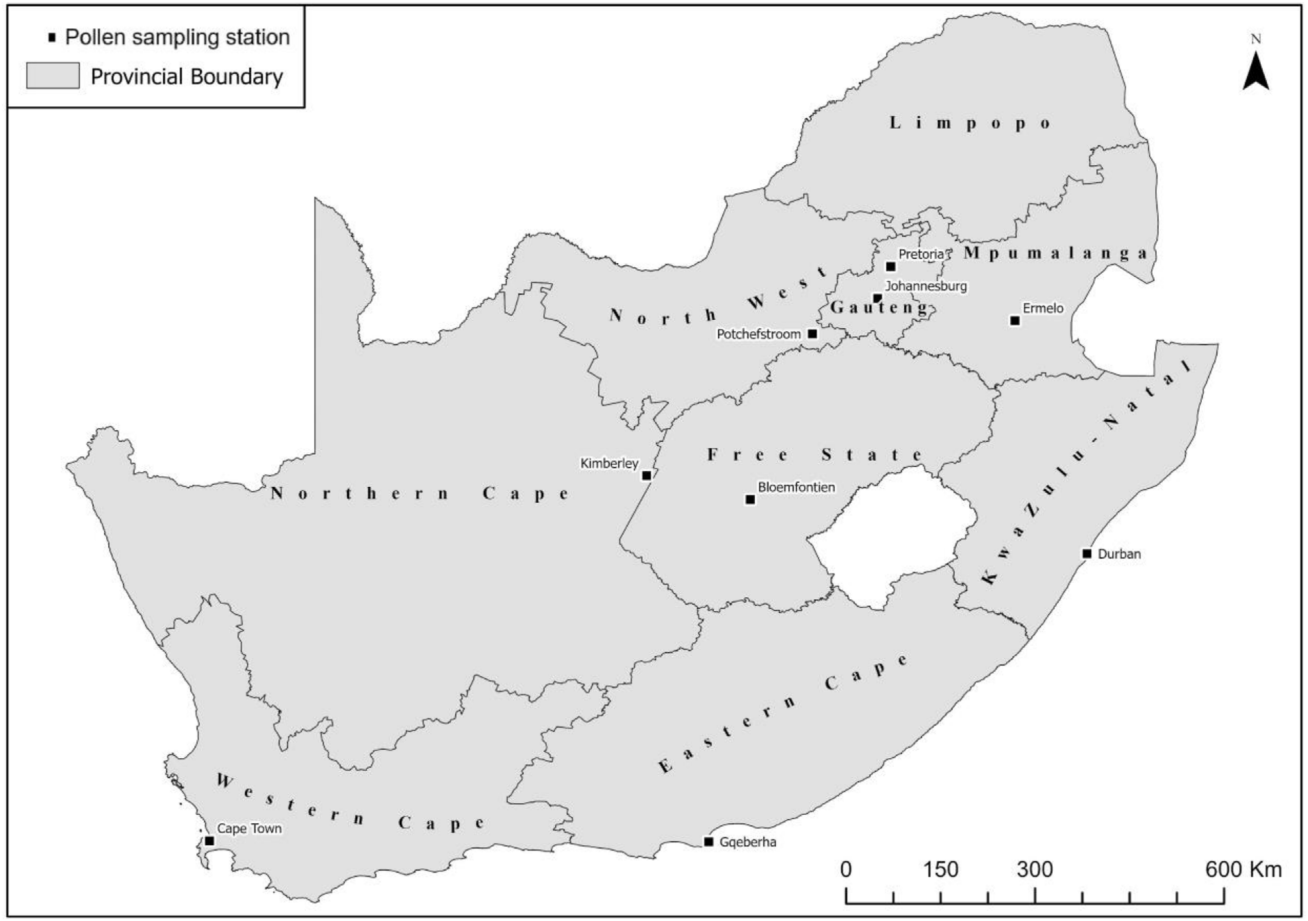
Location of spore traps in South African cities

Daily mean pollen concentrations of *Ambrosia* were recorded between August 2019 and December 2023. Airborne pollen was recorded in 9 South African cities (Supplemental Table 1). These stations routinely collect airborne pollen from ambient air using a Burkard spore trap (Burkard Manufacturing, UK) based on the Hirst design.^28^ Fig. 1 shows the location of 9 sampling sites studied in South Africa. All spore traps are placed c. 15–20 m above ground level on roofs of faculties, research centres, and hospital buildings. The aerobiological sampling, samples preparation, pollen counting and data interpretation was performed following the minimum requirements from the European Society of Aerobiology.^29^ The annual pollen index (API expressed as pollen grains/m^3^; p.g/m^3^) defines the daily sum of *Ambrosia* pollen concentration per year. The peak day is defined as the day when the maximum daily mean concentration is reached in the air (peak value).^29^ The temporal occurrence of *Ambrosia* pollen per study site was examined and the number of days equal to or greater than 1 p.g/m^3^ was calculated.

To determine the degree of risk of allergy that may trigger hypersensitivity symptoms during *Ambrosia* pollen season, the number of days on which the pollen concentration equalled or exceeded the threshold of 5 p.g/m^3^ value was calculated following Taramarcaz et al.^30^

### Pollen sequencing using eDNA metabarcoding

#### Sample selection

Given that *Ambrosia* eDNA metabarcoding is a pilot study conducted by SAPNET, samples were selected only from 6 aerobiological monitoring stations Bloemfontein, Cape Town, Durban, Johannesburg, Kimberley, and Potchefstroom during the sampling period 2022–2023.

#### Pollen eDNA extraction, amplification, and sequencing

Collected matter contained within the second half of the strip was transferred into microcentrifuge tubes for eDNA extraction using the Nucleomag kit (Macherey–Nagel, Düren, Germany), as suggested by Leontidou et al.^31^ Impurities and potential inhibitors were removed using the OneStep PCR inhibitor removal kit (Zymo Research) and quantified following the method described by Prosser and Hebert.^32^ The amplification of taxonomic barcodes targeting the ribulose-1,5-bisphosphate carboxylase (rbcL) and internal transcribed spacer 2 (ITS2) and their subsequent preparation into DNA libraries for sequencing was conducted following the PCR method described by Brennan et al.^33^ with RBCLaf and RBCLr506^34^^,^^35^ and ITS-S2F and ITS4^36^^,^^37^ primer pairs (Supplemental Table 2). The final library pool was spiked with 10% PhiX to improve diversity and loaded onto an Illumina MiSeqV2(300 cycles) for sequencing.

#### Bioinformatic data processing

Sequence data were assessed for quality with FastQC v0.12.1 and analysed following the meta-barcoding dual indexing pipeline method described by Sickel et al.^38^ The sequence reads were classified using USEARCH and RPD classifier v2.10.2 algorithms with a minimum raw score of 20 for the first-level UTAX classification and a minimum bootstrap value of 0.85 for the first-level RDP classification.

### Diagnosis of ragweed pollen sensitisation

To evaluate the baseline sensitisation rates for *Ambrosia* pollen – an allergen not currently part of standard testing recommendations in South Africa, we conducted a retrospective audit of all patients that underwent the multicomponent IgE array - Allergy Xplorer (ALEX^2^) test between February 2019 and February 2024. Patients undergoing this test were presented to the allergy clinics in Cape Town, South Africa. Three *Ambrosia* allergens were tested Amb, Amb a1, Amb a 4. A result of >0.35 IU/ml is considered positive.

### Statistical analysis

A descriptive statistical analysis of the *Ambrosia* pollen database based on a daily concentration was performed using MS Excel 2024 and the statistical programming language SPSS version 2021 for Windows, which was executed to analyse the frequency and percentage for categorical variables.

## Results

### Spatial distribution of *Ambrosia* in South Africa from SA national botanical records

**Fig. 2:**
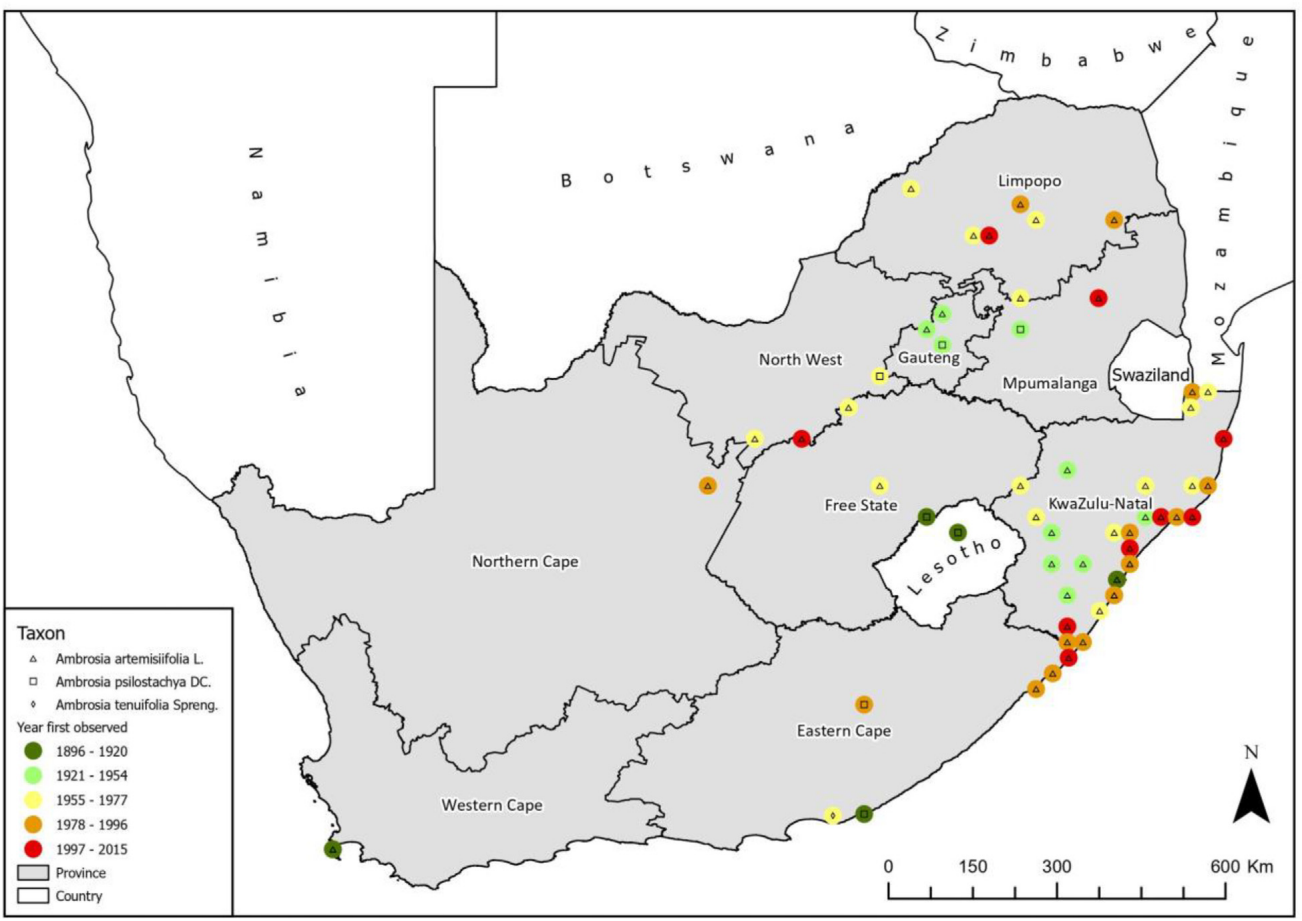
Map depicting the spread of *Ambrosia* spp. spread in southern Africa and Lesotho 1896–2015

The South African National Biodiversity Institute (http://posa.sanbi.org/sanbi/Explore) records were accessed to document the botanical records of the presence of *Ambrosia* spp. across South Africa. These records cover the period 1896 and 2015 (Fig. 2). *Ambrosia artemisiifolia* predominately is reported to occur in KwaZulu-Natal, Limpopo; *Ambrosia psilostachya* in North-West, Gauteng, Mpumalanga, Eastern Cape, and *Ambrosia tenuifolia* was recorded exclusively in the Eastern Cape province.

### New records of Ambrosia from SAPNET monitoring

*Ambrosia* pollen was recorded in 5 aerobiological monitoring stations: Durban, Potchefstroom, Kimberley, Pretoria, and Johannesburg during the monitored period (Table 1). Annual sums of *Ambrosia* pollen grains (API) varied between the 5 sites, ranked here from highest to lowest: Potchefstroom (API: 308 p.g/m^3^; 2022–2023), Durban (API: 66 p.g/m^3^; 2021–2022), Pretoria (API: 17 p.g/m^3^; 2022–2023), and Kimberley (API: 16 p.g/m^3^; 2022–2023). *Ambrosia* API showed a wide range at certain sites such as Kimberley, where the annual levels of *Ambrosia* pollen reached 16 p.g/m^3^ (2022–2023), compared to during the first 3 years of sampling, where pollen production was between 1 and 6 grains (spore trap location unchanged). In contrast, the Durban monitoring station recorded a minor annual variation with a maximum of 66 p.g/m^3^ (2021–2022) and a minimum of 26 p.g/m^3^ (2019–2020).

**Table 1:** Spatial characteristics of *Ambrosia* pollen in 5 sampling sites in South Africa.

Site		2019/2020	2020/2021	2021/2022	2022/2023
JHB	API	∗	∗	∗	1
	Peak conc. (p.g/m^3^)	∗	∗	∗	1
	Peak date	∗	∗	∗	17-04-23
	No. of days concentration ≥1 p.g/m^3^	∗	∗	∗	2
	No. of days concentration ≥5 p.g/m^3^	∗	∗	∗	0
KMB	API	6	1	1	16
	Peak conc. (p.g/m^3^)	2	1	1	5
	Peak date	13-04-20	20-03-21	10-04-22	13-08-22
	Nb of days concentration ≥ 1p.g/m^3^	6	2	2	10
	Nb.of days concentration ≥5 p.g/m^3^	0	0	0	1
PTA	API	∗	5	∗	17
	Peak conc. (p.g/m^3^)	∗	2	∗	3
	Peak date	∗	15-04-21	∗	12-08-22
	Nb of days concentration ≥1 p.g/m^3^	∗	5	∗	13
	No. of days concentration ≥5 p.g/m^3^	∗	0	∗	0
DBN	API	26	39	66	40
	Peak conc. (p.g/m^3^)	4	6	10	4
	Peak date	10-03-20	12-03-21	12-01-22	04-03-23
	Nb of days concentration ≥1 p.g/m^3^	19	23	35	28
	Nb of days concentration ≥5 p.g/m^3^	0	2	2	0
POT	API	∗∗	∗∗	∗∗	308
	Peak conc. (p.g/m^3^)	∗∗	∗∗	∗∗	47
	Peak date	∗∗	∗∗	∗∗	17-04-23
	Nb of days concentration ≥1 p.g/m^3^	∗∗	∗∗	∗∗	42
	Nb of days concentration ≥5 p.g/m^3^	∗∗	∗∗	∗∗	14

JHB: Johannesburg; KMB: Kimberley; PTA: Pretoria; DBN: Durban; BFN: Bloemfontein; POT: Potchefstroom. ∗JHB, KMB, PTA, DBN: Pollen not recorded; ∗∗Sampling has not started.

**Fig. 3:**
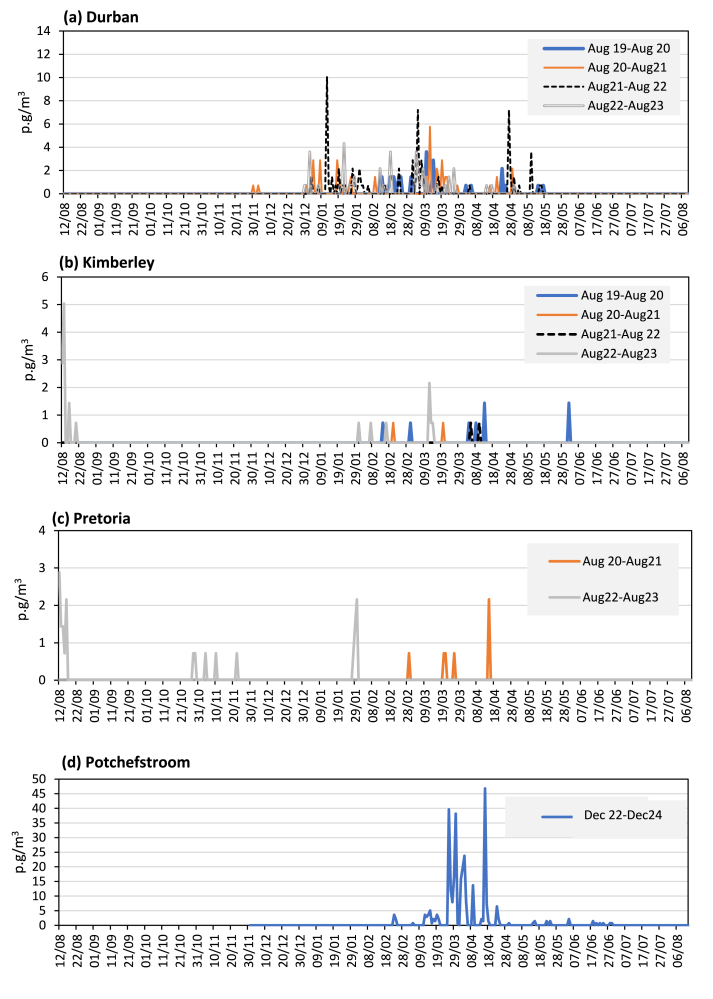
Fluctuations of *Ambrosia* pollen in the aerobiological sampling stations during the studied period. **a.** Durban, **b**. Kimberley, **c**. Pretoria, **d.** Potchefstroom

Over the studied years, the number of days where *Ambrosia* pollen was ≥1p.g/m^3^ ranged from just 2 days in Johannesburg to 42 days in Potchefstroom. In Durban, *Ambrosia* pollen was detectable in the air during a range of 19 days (2019–2020) and 35 days (2021–2022), in Kimberley between 2 days (2020–2022) and 10 days (2022–2023), and in Pretoria 5 days (2020–2021) and 13 days (2022–2023). Low daily mean concentrations were recorded at Johannesburg, Kimberley, Pretoria, and Durban with the highest values of 1, 5, 3, and 10 p.g/m^3^, respectively, compared to Potchefstroom, with a peak concentration of 47 p.g/m^3^ (Table 1, Fig. 3). The timing of peak days varies between the sampling years and the stations. For the 4 years, the earliest peak day was 12 January (Durban, 2021–2022) and the latest was 13 August (Kimberley, 2022–2023).

The number of days with threshold concentrations equal to or exceeding the limit of a daily average of 5 p.g/m^3^ at which individuals allergic to *Ambrosia* pollen might have allergic symptoms was recorded. The highest value was in Potchefstroom (14 days) and the lowest was in Durban (2 days). No risk days were observed for the other sampling sites.

Fig. 3 shows the seasonal variation of ragweed pollen at the 4 monitoring stations (2019–2023) with high API's. Daily mean concentration varies from year to year and depends on the location of the aerobiological station (Table 1, Fig. 3). All curves representing the *Ambrosi*a pollen season pattern showed the presence of several peaks. In Durban, *Ambrosia* pollen is present in the atmosphere from the end of December until the middle of May. Small peaks are observed with a maximum of daily mean concentrations ranging between 1 and 4 p.g/m^3^. However, 3 abundant peaks are observed in January 2020, March 2020, and April 27, 2020, with a maximum of 10, 7 and 7 p.g/m^3^, respectively. In Kimberley, low pollen counts are detected from the end of January until the middle of April, (maximum ranging of 1-2 p.g/m^3^), except for an early peak observed in August 2022–2023. In contrast, a short-term flowering period has been noted at Potchefstroom sampling station. Significant 3 peaks ≥30 p.g/m^3^ are observed between the March 23 and April 19, although in contrast to other sites, this represented just 1 season of data. In Pretoria, *Ambrosia* pollen grains are noted on several days during the studied period, in October–November, and January–April.

### *Ambrosia* speciation using metabarcoding

We identified 2 species - *Ambrosia artemisiifolia* and *Ambrosia trifida* using eDNA metabarcoding. *Ambrosia artemisiifolia* was identified in samples from Bloemfontein, Cape Town, and Potchefstroom. In Johannesburg and Durban, both *Ambrosia artemisiifolia* and *Ambrosia trifida* were identified.

### Clinical data of *Ambrosia* aeroallergen testing

**Fig. 4:**
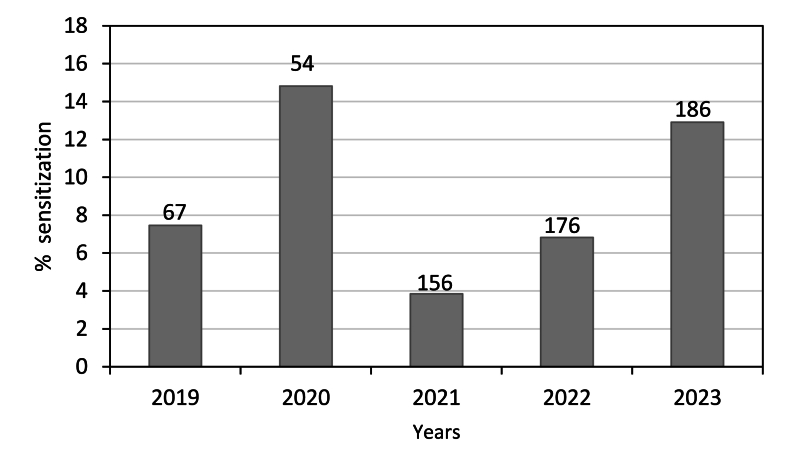
The Number of Alex^2^ tests performed in Cape Town and percentage of overall positive rates of patients sensitized for Ambrosia pollen per year

**Fig. 5:**
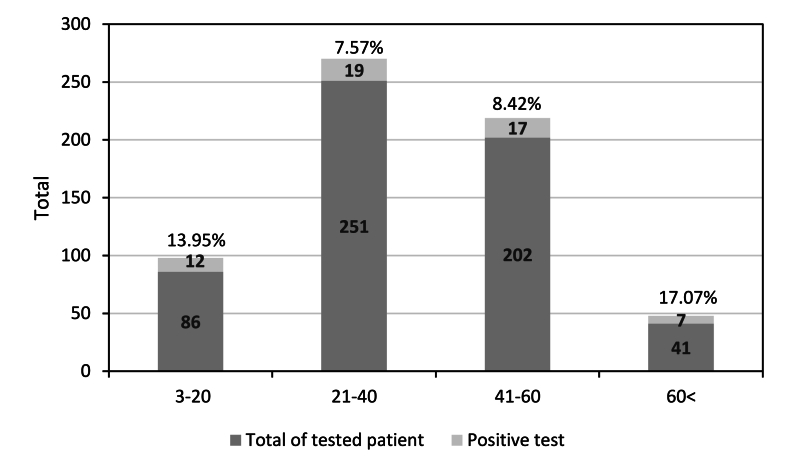
Total of performed Alex^2^ test to *Ambrosia* pollen, number and percentage of positive test among different age group in Cape Town

Out of 673 tested patients, 55 patients were found to be sensitized to *Ambrosia* allergens with an overall rate of 8.2%. The number of Alex^2^ tests performed and the percentage of patients with a positive IgE over 5 years are shown in Fig. 4. The analysis shows that the percentage sensitisation to *Ambrosia* pollen was low (3.84% (2021)-14.81% (2020)). The general pattern of patients sensitized was higher in the group aged between 21 and 40 years than in those aged 41–60, 3–20 or above 60 years (19 vs. 17, 12, and 7, respectively) (Fig. 5).

## Discussion

Many botanical studies have demonstrated the presence of 3 allergenic *Ambrosia* species: *Ambrosia artemisiifolia*, *Ambrosia tenuifolia*, and *Ambrosia psilostachya* in South Africa.^18^ However, this is the first aerobiological documentation of any *Ambrosia* spp. in South Africa, and up until this point it has not been considered an allergen of importance. *Ambrosia* spp. pollen grains were initially identified during a routine microscope analysis of the aerospora samples collected from Durban and Kimberley in 2021, Pretoria in 2021, and Potchefstroom and Johannesburg in 2023. Since this first confirmed observation of *Ambrosia* pollen, attention and awareness of the distribution and amounts of *Ambrosia* pollen traits variation, their species source origin and potential health impact on the population of major cities in South Africa has been initiated by aerobiologists, allergists, and urban ecologists.

In South Africa, maximum emissions of *Ambrosia* pollen were noted in the late summer months, starting towards the end of January, and continuing until April with some fluctuations in Kimberley, Durban, and Pretoria. A short but pronounced period of *Ambrosia* pollen presence was noted in Potchefstroom, during late summer, between March and April. In many central European countries, *Ambrosia* was the most important allergen plant for sensitized patients during the late summer between late July and mid-September.^2^^,^^7^

Results of the present study showed considerable fluctuation in ragweed pollen concentration across monitoring sites. The highest concentrations were measured in Potchefstroom, a city in the Grassland Biome.^39^ Extensive vegetation sampling of different land-use areas in Potchefstroom in the 1990s has shown *Ambrosia psilostachya* to be present in wetland areas where they dominated dry, overgrazed areas along the river courses.^40^ Since then, the species have been observed in degraded grasslands and rural habitats such as road verges, railway embankments and vacant lots. Lower *Ambrosia* pollen concentrations were recorded at the sampling sites of Johannesburg, Pretoria, Kimberley, and Durban. Our aerobiological observations match the herbarium records (SANBI; http://posa.sanbi.org/sanbi/Explore) (Fig. 2), which show the low presence of *Ambrosia* in Gauteng and Northern Cape, whereas the records of *Ambrosi*a spp. in the provinces of KwaZulu-Natal and Eastern Cape were high. Aerobiological monitoring notes that after release, air pollen can be transported between 10 and 1000 km,^8^ meaning that the lower pollen counts recorded at these study sites may also be indicative of long-distance transport between the pollen source and the sampler. The long-range pollen transport and distance between the source and sampler may have an important effect on the occurrence of days with *Ambrosia* pollen and variation of pollen concentrations.^8^ Furthermore, the pronounced *Ambrosi*a pollen peak flowering pattern variations can be linked to the ecological features and phenology of *Ambrosia*. Similar patterns were observed in Moscow,^41^ Catalonia,^42^ and Britain,^43^ with multiflowering peaks characterizing the taxonomic species composition of airborne *Ambrosia* pollen over the flowering time period.

With regards to the qualitative assessment of *Ambrosia* pollen using eDNA metabarcoding, *Ambrosia* *artemisiifolia* was identified from samples of Bloemfontein, Cape Town, and Potchefstroom. In Johannesburg and Durban, *Ambrosia artemisiifolia* and *Ambrosia* *trifida* were found. From the 3 species identified in South Africa based on botanical surveys, ie, *Ambrosia artemisiifolia, Ambrosia psilostachya,* and *Ambrosia tenuifolia*, only *Ambrosia artemisiifolia* was confirmed with DNA metabarcoding, a species not previously observed in South Africa. This proof of concept work focused on *Ambrosia* pollen, using eDNA, represents the first confirmation of *Ambrosia artemisiifolia* species in South Africa from aerobiological sampling. The inconsistencies between South African National Biodiversity Institute (SANBI) species records, microscopic count, and species assignments derived from eDNA metabarcoding data, are likely a result of the low number of aerobiological samples used for this initial work (ie, 3 samples per site and 2 samples for Potchefstroom). Further work is planned to more comprehensively use eDNA metabarcoding to map *Ambrosia* species diversity across aerobiological samples.

Various countries have documented the importance of ragweed pollen as an aeroallergen.^1^ Our study found a very low rate of *Ambrosia* sensitisation pattern amongst allergy patients presenting for testing in Cape Town, South Africa. Notably, no *Ambrosia* spp. were detected in the Cape Town spore trap during this monitoring period, although low concentrations of *Ambrosia* were detected in a Cape Town sample using eDNA metabarcoding. Therefore, this likely represents a background sensitisation rate as a result of human movement. Sensitisation rates are very high in countries where *Ambrosia* spp. are well established and pollen monitors show high counts, including 80% in Budapest, Hungary,^2^ 70% in Lugano, Milano,^44^ 70% in France, Czech Republic, and Austria, and 17% in southern Switzerland.^45^ There are several suggestions regarding the threshold value of ragweed pollen triggering allergy symptoms. Banken and Comtois^46^ mention that symptoms should be expected with a level of 5–10 p.g/m^3^, whilst Jäger^47^ reported a threshold value for clinical symptoms for sensitive patients as 20 p.g/m^3^. In our study, we considered the critical threshold values of daily ragweed pollen concentration for provoking symptoms in sensitized patients equal to or above 5 p.g/m^3^ air. Fortunately there were a few days where symptom trigger thresholds were reached during the monitoring periods. Further studies of sensitisation patterns across other parts of South Africa are now required, and an important outcome of this work is to make allergists aware of the need to recognise *Ambrosia* spp. as a potential key late summer aeroallergens, particularly in northern inland and coastal areas of South Africa. These baseline rates from an area without environmental *Ambrosia* exposure will serve as a useful reference point to track expansion and any increase in *Ambrosia* sensitisation rates.

Data from Northern Hemisphere modelling suggests massive increases in new sensitisations to *Ambrosia* due to increasing global temperature leading to the growth of suitable habitats.^7^ This study, ongoing monitoring and mapping of sensitisation patterns in the coming 5 to 10 years will be imperative to track the spread of *Ambrosia* and sensitisation patterns in South Africa. Furthermore, from an urban planning and management perspective, we advise that control measures and effective strategies need to be adopted against *Ambrosia* to reduce the spread of invasive plant populations and decrease the atmospheric pollen load.

The uncontrolled spread of this invasive weed in the country could represent a serious threat to human health. An important first step in managing these alien invasive species is raising awareness among land managers particularly municipal authorities of the Eastern part of South Africa and landscape specialists. Eradication of *Ambrosia* in urban ruderal areas will be challenging as they are annual species and their occurrence and population dynamics need to be closely monitored. We further suggest that the management of *Ambrosia* should form a component of urban green infrastructure management practices. In addition, ecological restoration of the grasslands vegetation type, for example by planting native species will contribute towards the long-term sustainability of species diversity and may reduce the available habitats for the invasion of alien species,^48^ such as *Ambrosia*.

## Conclusion

Our aerobiological monitoring data confirm the historical scenarios from other continents of the spectacular invasion process of *Ambrosia*. Through the aerobiological monitoring carried out from 2019 by SAPNET, quantitative and qualitative information on the distribution of *Ambrosia* was obtained. The occurrence of ragweed pollen is evidence of the presence of *Ambrosia* species in South Africa. A significant fluctuation was associated with a noticeable discontinuity of total pollen and daily mean concentrations of ragweed grains in the air of 5 South African cities during 4 sampling years. The strong occurrence of *Ambrosia* pollen in Potchefstroom/North-West province is in good agreement with the distribution pattern of the genus in South Africa. It points to the prevalence of the taxon in northeastern South Africa in the summer rainfall region, eg, in the provinces Mpumalanga and Limpopo, which are currently monitoring gaps in SAPNET. Long-term aerobiological monitoring, combined with increased testing for sensitisation across diverse urban and rural communities is necessary to understand the comprehensive and prolific growth of ragweed plants in other cities of Southern Africa.

## Abbreviations

API, Annual sums of pollen grains; ALEX^2^, multicomponent IgE array - Allergy Xplorer; P.g/m^3^, pollen grains/m^3^; SAPNET, South African Pollen Network; SANBI, South African National Biodiversity Institute; SA, South Africa.

## Availability of data and materials

The datasets used during the present study are available from the corresponding author upon reasonable request.

## Author contributions

**Dorra Gharbi:** Conceptualization; design, data acquisition; data analysis; Formal analysis, visualization; writing-original draft; writing- review & editing. **Dilys Berman:** Conceptualization; project administration; data acquisition; writing – original draft; writing – review & editing. **Frank H. Neumann:** Conceptualization; investigation; writing – original draft; writing – review & editing. **Trevor Hill:** Conceptualization; writing – original, visualization, draft; writing – review & editing. **Siyavuya Sidla:** Data analysis; writing. **Sarel S. Cilliers:** investigation; visualization; writing original draft; writing; review & editing. **Jurgens Staats:** writing original draft; writing; review & editing. **Nanike Esterhuizen:** project administration; data acquisition; review & editing. **Linus Ajikah:** data acquisition; writing original draft. **Moteng E. Moseri:** data acquisition; review & editing. **Lynne J. Quick:** data acquisition; visualization; writing original draft; review & editing. **Erin Hilmer:** data acquisition; project administration; writing – review & editing. **Andri Van Aardt:** data acquisition; project administration; writing – original draft; writing – review & editing. **Juanette John:** project administration. **Rebecca Garland:** data acquisition; writing – original draft writing – review & editing. **Jemma Finch:** Writing – original draft; writing – review & editing. **Werner Hoek:** Investigation; project administration writing – original draft. **Marion Bamford:** Conceptualization writing – review & editing. **Riaz Y. Seedat:** writing – review & editing. **Ahmed I. Manjra:** writing – review & editing. **Jonny Peter:** Conceptualization; funding acquisition; supervision; writing – original draft; writing – review & editing.

## Ethics approval

This study protocol was approved by the Faculty of Health Sciences Human Ethics Committee (HREC REF: 368/2024).

## Authors consent for publication

We confirm that the manuscript has been read and approved by all named authors and that there are no other persons who satisfied the criteria for authorship but are not listed. We further confirm that all have approved the order of the authors listed in the author's manuscript. We confirm that we have given due consideration to the protection of intellectual property associated with this work and that there are no impediments to publication, including the timing of publication, concerning intellectual property. In doing so, we confirm that we have followed the regulations of our institutions concerning intellectual property. We further confirm that any aspect of the work covered in this manuscript that has involved human patients has been conducted with the ethical approval of all relevant bodies and that such approvals are acknowledged within the manuscript. We understand that the corresponding author is the principal contact for the editorial process. He is responsible for communicating with the other authors about the progress, submission of revisions, and the final approval of proofs. We confirm that we have provided a current, correct corresponding email address.

## Funding

We received financial support from UCT Faculty of Health Sciences Postdoctoral Research Fellow Award (Dorra Gharbi), The Real Pollen Count received industry sponsorship from Cipla, Clicks, Dr. Reddy's, Thermo Fisher Scientific, Novartis, Glenmark, SA Natural Products, and Twinsaver.

## Declaration of competing interest

The authors have declared that no competing interests exist.

## Footnotes

Appendix A Supplementary data to this article can be found online at https://doi.org/10.1016/j.waojou.2024.101011.
