# Supplementary material for: Ambrosia (ragweed) pollen — A growing aeroallergen of concern in South Africa
Source: World Allergy Organ J. 2024 Dec 2;17(12):101011. doi: 10.1016/j.waojou.2024.101011 (PMC11652763; doi:10.1016/j.waojou.2024.101011)
Supplement: Multimedia component 1 [file mmc1.docx]

Supplementary table S1. Geographical, meteorological characteristics and aerobiological samplers’ coordinates of the study cities (adopted from Esterhuizen et al. 2023).

| City | Province | Sampler coordinates | Biomes | Köppen Climate | Average  Rainfall | Average  Temperatures  (Tm- TM) |
| --- | --- | --- | --- | --- | --- | --- |
| CPT | Western Cape | 33°57'12"S 18°29'11"E | Fynbos | Mediterranean | Winter 515 mm | 11.4 °-22.0 °C |
| JHB | Gauteng | 26°11'34.8"S 28°01'48.0"E | Grassland | Subtropical | Summer 713 mm | 10.1 °C- 21.9 °C |
| PTA | Gauteng | 25°54’42 64”S, 28°12’38.22”E | Savanna | Subtropical | Summer 673 mm | 12.3 °C- 24.7 °C |
| BFN | Free State | 29° 6'36.32"S 26°11'6.79"E | Grassland | Semi-arid | Summer 559 mm | 7.5 °C- 24.4 °C |
| KMB | Northern Cape | 28° 44' 30.9948'' S 24° 46' 18.9984'' E | Savanna | Semi-arid | Summer 414 mm | 10.9 °C- 26.1 °C |
| DBN | KwaZulu-Natal | 29°51'03"S 30°55'59"E | Indian Ocean  Coastal Belt | Humid-subtropical | Summer 1019 mm | 16.5 °C-25.2 °C |
| PE | Eastern Cape | 34° 0'4.69"S 25°40'2.07"E | AlbanyThicket | Oceanic | Year-round 624 mm | 13.5 °C-22.3 °C |
| Potch | North-West | 26°41'21"S 27°05'27"E | Grassland | Semi-arid | Summer 600 mm | 0 °C-30 °C |
| Ermel | Mpumalanga | 26°29'48"S 29°59'07"E | Grassland | Subtropical | Summer 1100 mm | 0 °C-30 °C |

*Mean daily temperature (Tm = minimum temperature; TM = maximum temperature); CPT: Cape Town; JHB: Johannesburg; PTA: Pretoria; BFN: Bloemfontein; KMB: Kimberley; DBN: Durban; PE: Gqeberha (previously Port Elizabeth); Potch: Potchefstroom; Ermel: Ermelo.
