# Supplementary material for: Ambrosia (ragweed) pollen — A growing aeroallergen of concern in South Africa
Source: World Allergy Organ J. 2024 Dec 2;17(12):101011. doi: 10.1016/j.waojou.2024.101011 (PMC11652763; doi:10.1016/j.waojou.2024.101011)
Supplement: Multimedia component 2 [file mmc2.docx]

Supplementary table S2. Primer pairs used for *Ambrosia* pollen eDNA sequencing.

| PrimerID | Primer sequence (5’ 🡪3’) |
| --- | --- |
| *rbcLaf* | **ACACTCTTTCCCTACACGACGCTCTTCCGATCT**NNNNNNATGTCACCACAAACAGAAAC |
| *rbcLr506* | **GTGACTGGAGTTCAGACGTGTGCTCTTCCGATCT**AGGGGACGACCATACTTGTTCA |
| *ITS-2F* | **ACACTCTTTCCCTACACGACGCTCTTCCGATCT**NNNNNATGCGATACTTGGTGTGAAT |
| *ITS-4R* | **GTGACTGGAGTTCAGACGTGTGCTCTTCCGATCT**TCCTCCGCTTATTGATATGC |

*Sequences highlighted in bold are universal primer tails
